# Oxy-Phosphate of Zinc

**Published:** 1883-04

**Authors:** E. G. Betty


### ARTICLE V.

### *Oxy-Phosphate of Zinc.*

BY E. G. BETTY, D. D. S.

One of the most useful materials in the daily practice of the operator is the oxy-phosphate of zinc. So useful and reliable is it, that it has quickly superceded the oxy-chloride, formerly so greatly in demand to meet the requirements of just such an article. As the material is comparatively new, and its manipulation somewhat different from that of the chloride, it may not be out of place to hazard a few suggestions in regard to its manipulation and uses. The properties depend, of course upon the chemical process that takes place when the powder, oxide of zinc, and the solution of phosphorous acid, are mixed together. This change, whether it is solely chemical, or an obscure process of crystallization, or a combination of the two, one following the other, is beyond the province of this paper. That question is left for the chemist, who is welcome, at any time, to give us the result of his investigations. The intention is merely to outline the practical; that, to most operators, is, after all, of the most value. So far as as the mixing of the oxy-phosphate is concerned, it may be laid down as a rule, that the powder is in all cases to be added to the solution. The required stiffness or flaccidity of the mixture is to be governed by the amount of powder added to the liquid. After repeated trials, and an aggravating experience, this method has been found to give the most satisfactory results. In very large cavities, not encroaching too near the pulp, when the intention is to fill with gold at the same sitting, it is desirable to guard against the shock of violent and sudden thermal changes. As a barrier between the metal and the dentine, it subserves a good purpose. Should the dentine be very sensitive, the oxy-phosphate is better made stiff and quickly pressed into place with a suitable burnisher. When mixed stiff the affinities of the base and the acid are so nearly or completely satisfied that there does not remain upon the surface of the plastic bolus sufficient acid to produce the sudden and acute pain that so many patients complain of. This pain, which so many operators make the ground of their objection to the use of the oxy-phosphate, is due to two different causes: First, when the material is made thin the acid predominates and immediately attacks the sensitive surface of the dentine in the cavity. Second, even though the phosphate be made stiff, it may at the same time be so far below the tooth in temperature that, when introduced, it will cause pain by immediately absorbing heat from the tooth. This is reasonable to suppose, because the cavity is dry and the bolus (if it may be termed,) is wet and of lower temperature. In the first instance the pain may be avoided, to a considerable degree, by previously lining the cavity with the dry powder. The thin mixture can then be poured in safely, the powder receiving and combining with the free acid, thus protecting the dentine. Should the cavity be in the upper jaw, the thin mixture can, with little difficulty, be flowed in by first touching some interior point of the cavity with a small quantity of it. The bulk once touching this point of attraction, will readily flow into place. If made thin, the phosphate will necessarily require more time to set and become hard enough to withstand the percussion of the mallet. In the second case the materials ought to be prepared on a heated surface, to raise the temperature of the mass near that of the tooth. This is simply done by mixing it on the bottom of a tumbler previously containing hot water, or upon a square of glass or porcelain that has been warmed near the stove. The addition of heat to the mass, however, will hasten the setting, and it will be found necessary either to add less power to the liquid, or be very, very expeditious in introducing it into the tooth. A little close observation will enable the operator to determine just the required consistency, and the rapidity of crystallization when mixed warm.

The adhesion of the mass to the instruments while handling it, is very annoying to those who are too lazy to slightly oil the instrument before using it. Instead of being an objection, this very adhesiveness is a desirable quality, and often serves us well when the cavity is of poor retaining shape, and we wish to fill temporarily. In such cases the adhesion will be found greatest when the mass is mixed thin. For capping an exposed pulp successfully there is probably nothing better than the oxy-phosphate, if it is properly handled. In the estimation of the writer, many failures are due to reckless excavation, and the consequent pain to which the pulp and surrounding dentine are subjected. The less pain attending the capping of the pulp, the greater will be the chances of ultimate success. After the excavation is completed the pulp can be covered with a thin skin of gum by flowing over it a little of the compound tincture of benzoin. The walls of the cavity may also be coated with it, a few moments only being required for the evaporation of the alcohol. It may be expedited with the warm air current, gently and gradually applied. The covering of gum may be thickened, if desirable, by two or three applications of the tincture at short intervals. A small quantity of the phosphate may now be mixed thin, on a warmed surface, and flowed directly over the exposure, allowing it to run over the edges so that it will bear upon the solid dentine. When hardened the capping may be trimmed with an excavator. The cavity can now with safety be filled as an ordinary one, without fear of producing pressure on the pulp. It is best to fill with a stiff bolus of the phosphate and allow it to remain for a year or more, as the material is good for that length of time in the majority of mouths; longer in some. By proceeding in the manner above detailed, the operator will avoid producing that "shock" to the pulp that is caused either by placing in direct contact with it an irritating acid, or suddenly reducing its temperature. If the pulp is outraged by careless handling and its sensibility subjected to a severe trial, we cannot hope for, much less expect, it to recover its wonted functions. It may not be generally known, but it is nevertheless a fact, that a violent toothache, due to an exposure, may be almost instantly controlled by an application of the compound tincture of benzoin. It was this fact that suggested to the writer the propriety of using it as a preliminary covering for the pulp; and experience has proved it very efficacious in many instances. It also serves very well when applied to the dentine over and around the pulp, during excavation, taking care not to flood the cavity with it while cold. The pledget of cotton saturated with it can be warmed over the lamp. The soothing effect of this tincture may be due, in some degree, to a slight anodyne property of some of its ingrediments. It is probably more likely that its effect is owing to the evaporation of the alcohol, leaving a film of sticky gum that completely protects the surface from the atmosphere. Be that as it may, it is well worthy of a trial, and will not be found ungrateful. However, it was not intended to discuss the treatment of exposed pulps, and the digression may be pardoned on the ground that it is difficult to exclude it when considering the subject of this article.

Fossiline, the English preparation of the oxy-phosphate, (for it can scarcely be anything else,) that was introduced a year and a half ago into the United States, is a very good one. It requires some patience to obtain the best results from it, as it sets very rapidly. If mixed too stiff it will not bear much manipulation while introducing it, the tendency being to crumble or granulate, and, in consequence, losing its integrity as a mass. This, in fact, is true of all the oxy-phosphates, but is not necessarily an objection to it, for it can be guarded against. The phosphate is a very good material with which to fill very sensitive teeth, when it is desirable to postpone, indefinitely, the introduction of a metallic filling. For this purpose it is much superior to the gutta-percha fillings. After six months or a year, the greater portion of the phosphate can be removed and the metallic filling put in over the remainder. The sensitiveness will by this time have been greatly modified, or will have disappeared. A great deal can be said about this material and its many uses as a temporary filling for dead teeth, sensitive ones, exposed pulps, etc., with which the reader is familar, making it superfluous to repeat.—*Ohio State Journal of Dental Science.*

